# Cognitive function is associated with risk aversion in community-based older persons

**DOI:** 10.1186/1471-2318-11-53

**Published:** 2011-09-11

**Authors:** Patricia A Boyle, Lei Yu, Aron S Buchman, David I Laibson, David A Bennett

**Affiliations:** 1 Rush University Medical Center, Rush Alzheimer's Disease Center, 600 S. Paulina, Suite 1020, Chicago, IL 60612 USA; 2 Department of Behavioral Sciences, Rush Alzheimer's Disease Center, 600 S. Paulina, Suite 1020, Chicago, IL 60612 USA; 3 Department of Neurological Sciences, Rush Alzheimer's Disease Center, 600 S. Paulina, Suite 1020, Chicago, IL 60612 USA; 4 Harvard University, Department of Economics Littauer M-12, Harvard University, Cambridge, MA 02138 USA

## Abstract

**Background:**

Emerging data from younger and middle-aged persons suggest that cognitive ability is negatively associated with risk aversion, but this association has not been studied among older persons who are at high risk of experiencing loss of cognitive function.

**Methods:**

Using data from 369 community-dwelling older persons without dementia from the Rush Memory and Aging Project, an ongoing longitudinal epidemiologic study of aging, we examined the correlates of risk aversion and tested the hypothesis that cognition is negatively associated with risk aversion. Global cognition and five specific cognitive abilities were measured via detailed cognitive testing, and risk aversion was measured using standard behavioral economics questions in which participants were asked to choose between a certain monetary payment ($15) versus a gamble in which they could gain more than $15 or gain nothing; potential gamble gains ranged from $21.79 to $151.19 with the gain amounts varied randomly over questions. We first examined the bivariate associations of age, education, sex, income and cognition with risk aversion. Next, we examined the associations between cognition and risk aversion via mixed models adjusted for age, sex, education, and income. Finally, we conducted sensitivity analyses to ensure that our results were not driven by persons with preclinical cognitive impairment.

**Results:**

In bivariate analyses, sex, education, income and global cognition were associated with risk aversion. However, in a mixed effect model, only sex (estimate = -1.49, standard error (SE) = 0.39, p < 0.001) and global cognitive function (estimate = -1.05, standard error (SE) = 0.34, p < 0.003) were significantly inversely associated with risk aversion. Thus, a lower level of global cognitive function and female sex were associated with greater risk aversion. Moreover, performance on four out of the five cognitive domains was negatively related to risk aversion (*i.e*., semantic memory, episodic memory, working memory, and perceptual speed); performance on visuospatial abilities was not.

**Conclusion:**

A lower level of cognitive ability and female sex are associated with greater risk aversion in advanced age.

## Background

Compelling economics, behavioral economics, and neuroeconomics studies have shown that risk preferences are predictive of several real world outcomes, including economic outcomes, financial and healthcare decisions, and even health behaviors [1-4]. For example, individuals who are relatively risk averse tend to invest more conservatively (*e.g.*, invest the bulk of their savings in "safe" options such as Treasury bonds instead of higher yielding asset classes like stocks). Individuals who are risk averse also are less likely to engage in dangerous health behaviors such as cigarette smoking, excessive alcohol use, and seatbelt non-use [3,4]. Recognition of the important role of risk aversion has generated considerable interest in understanding its basis, and studies of younger and middle-aged persons have revealed several insights. Perhaps most importantly, these data suggest that something akin to intellectual or cognitive ability (*e.g.*, performance on the SATs or educational attainment tests) appears to be a robust correlate of risk aversion [5,6]. Notably, however, demographic factors such as age, education, and sex, and contextual factors such as income also are associated with risk aversion as well as cognition, making it difficult to disentangle the effects of cognition from demographic and contextual factors [5]. To date, we are not aware of studies that have attempted to measure the extent to which cognitive ability is related to risk aversion among community-based older persons after adjusting for relevant demographic and contextual factors.

Knowledge of the determinants of risk aversion in aging may have major financial and public health implications. Aging is widely regarded as a time of cognitive and functional decline, yet in the midst of the challenges of aging, older persons are confronted with a host of complex and risky decisions that arguably have as much (if not more) significance as decisions made earlier in life. For example, wealth tends to accumulate over a lifetime, and a large portion of the nation's capital is held and ultimately will be distributed by older persons. Allocation of resources, including investment choices, intergenerational transfers, philanthropic gifts, and even personal spending is inherently risky yet critically important for maximizing both individual well-being and prosperity for future generations. Moreover, unlike younger persons, older persons do not have the benefit of many years ahead in the workforce and opportunities to overcome financial mistakes. Similarly, older persons suffer a disproportionate burden of illness and are faced with some of the most challenging healthcare decisions (*e.g*., whether to undergo invasive treatments for conditions unlikely to be cured, end of life care), and their choices are associated with major personal, familial, and societal costs. Given the dramatic relative increase in the population of older adults and the implications of the financial and other decisions older people make, it is important that we understand older persons' risk preferences.

In this study, we examined the correlates of risk aversion among older persons and tested the hypothesis that cognitive ability is negatively related to risk aversion even after adjusting for relevant demographic and contextual factors; this pattern has been observed in relatively younger populations [6,7]. The current study analyzed the choices of community-based older adults from the Rush Memory and Aging Project, all of whom were free of dementia [8]. All underwent detailed cognitive evaluations and assessments of risk aversion using standard behavioral economics questions in which participants were asked to choose between a guaranteed payment of $15 or a gamble in which they could either gain various sums greater than $15 or nothing at all. We first examined the bivariate associations of age, sex, education, income, and cognition with risk aversion. Next, we examined the association of cognitive function, including global cognition and five specific domains (*i.e.*, semantic memory, episodic memory, working memory, perceptual speed, and visuospatial abilities), with risk aversion via mixed effect models adjusted for age, sex, education, and income. Finally, we conducted sensitivity analyses to ensure that our results were not driven by the inclusion of persons at the lowest end of cognitive ability.

## Methods

### Participants

Participants were 369 individuals from the Rush Memory and Aging Project, an ongoing longitudinal clinical-pathologic study of common chronic conditions of old age [8]. Study participants are residents of approximately 40 senior housing facilities in the Chicago metropolitan area, including subsidized housing facilities, retirement communities, and retirement homes. Participants in the Rush Memory and Aging Project undergo risk factor assessment, detailed annual clinical evaluations (see below), and organ donation at the time of death. The Memory and Aging Project was approved by the Institutional Review Board of Rush University Medical Center, and informed consent was obtained from each participant following a detailed presentation of the risks and benefits associated with study participation. Notably, however, the Memory and Aging Project began in 1997 and is ongoing, and the assessment of risk aversion began as part of a substudy on neuroeconomics initiated in 2008. This substudy also was approved by the Institutional Review Board of Rush University Medical Center, and participants in the neuroeconomics substudy completed a separate informed consent following a detailed presentation of the risks and benefits associated with participation in this substudy.

At the time of these analyses, there were a total of 939 living participants who were potentially eligible for risk aversion assessment; of those, 645 were approached, 427 participated, and 218 refused, rendering a participation rate of 66%. Of the 427 participants that completed their baseline interview including measurement of risk aversion, 39 were excluded due to dementia and 19 did not have complete data. This left a final sample of 369 persons eligible for these analyses; their characteristics are reported in Table 1.

**Table 1:** Characteristics of participants

Characteristic	Mean, SD, Range (or %)
Age	83.2 (6.7, 60.0-98.8)
Female Sex	276 (74.8%)
Education	15.1 (3.0, 7-28)
Income	
< $25K	99 (26.8%)
$25K-$50K	145 (39.3%)
> $50	125 (33.9%)
Global cognition	0.28 (0.51, -1.28-1.59)
MMSE	28.1 (2.0, 13.9-30)
Risk aversion	0.39 (0.31, 0.03-0.86)

### Clinical and cognitive evaluation

Details of the clinical evaluation have been described previously [8]. Briefly, each participant underwent a uniform structured evaluation, including medical history interviews, complete neurological evaluations and neuropsychological examinations. Cognitive function was assessed via a battery of 21 tests, including the MMSE [9], but MMSE scores were used only to describe the cohort. Scores on 19 tests are used to create summary indices of global cognitive function and five specific cognitive domains: episodic memory, semantic memory, working memory, perceptual speed, and visuospatial ability. Episodic memory is assessed via seven tests: immediate and delayed recall of story A from Logical Memory, immediate and delayed recall of the East Boston Story, Word List Memory, Word List Recall, and Word List Recognition; semantic memory is assessed via three tests: a 15-item version of the Boston Naming Test, Verbal Fluency, and a 15-item reading test; working memory is assessed via three tests: Digit Span Forward, Digit Span Backward and Digit Ordering; perceptual speed was assessed via four tests: Symbol Digit Modalities Test, Number Comparison, and two indices from a modified version of the Stroop Neuropsychological Screening Test; and visuospatial abilities are assessed via two tests: a 15-item version of Judgment of Line Orientation and a 16-item version of Standard Progressive Matrices. One additional test, Complex Ideational Material, is used for diagnostic classification purposes only.

To compute the composite measure of global cognitive function, raw scores on each of the individual tests were converted to z-scores using the baseline mean and standard deviation of the entire cohort, and the z-scores of all 19 tests were averaged [10,11]. Summary scores for five cognitive domains (*i.e*., episodic memory, semantic memory, working memory, perceptual speed, and visuospatial abilities) were derived by converting raw scores on each of the individual tests to z-scores using the mean and standard deviation of the entire cohort and then averaging the z-scores from tests within a specific domain. Psychometric information on these summary scores, including factor analytic support for the five domains, is contained in previous publications [11].

### Assessment of risk aversion

Risk aversion was assessed via a series of 10 questions used in standard behavioral economics approaches. Specifically, participants were asked, "Would you prefer $15 for sure, OR a coin toss in which you will get $[an amount greater than $15] if you flip heads or nothing if you flip tails?" Potential gamble gains ranged from $21.79 to $151.19 with the gain amounts varied randomly over questions. It is noteworthy that, when the potential gamble reaches $30.00, both the safe payment and the gamble have the same long run average or expected value. However, when the potential gamble gain exceeds $30, the expected value of the gamble exceeds the value of the safe payment.

### Clinical diagnoses

Clinical diagnoses were performed using a three stage process, as previously described [8]. First, neuropsychological tests were administered by trained technicians, scored by a computer, and ratings of impairment were assigned based on education-adjusted cut-off scores on 11 cognitive tests commonly used in the assessment of cognition in old age. Second, an experienced neuropsychologist, blinded to subject age, sex, and race, reviewed the results of the cognitive testing including impairment ratings, data on education, sensory and motor deficits, and rendered a clinical judgment regarding the presence of cognitive impairment. Third, diagnostic classification was performed by an experienced clinician blinded to all previously collected data after a review of all available data from that year's clinical evaluation, including the ratings by the neuropsychologist and the details of the neurological examination. The clinician then specified whether the participant met clinical criteria for dementia and probable AD recommended by the joint working group of the National Institute of Neurologic and Communicative Disorders and Stroke and the Alzheimer's Disease and Related Disorders Association, which require evidence of cognitive decline in memory and at least one other domain of cognitive function [12]. The diagnosis of mild cognitive impairment (MCI) was rendered for individuals who were found to have cognitive impairment by the neuropsychologist but who, in the judgment of the examining clinician, did not meet criteria for dementia. Persons who did not meet criteria for mild cognitive impairment or dementia, as determined by the clinician's review of all available data, were classified as having no cognitive impairment (NCI).

### Other covariates

Other variables used in the analyses included age (based on date of birth and date of cognitive testing), sex (females coded as 0 and males as 1), education (years of schooling completed), and income. Income was measured using the show card methodology, as previously described [8]; self reported annual income was ranked according to 10 possible categories: 1: $0-$4999, 2: $5000-$9999, 3: $10000-$14999, 4: $15000-$19999, 5: $20000-$24999, 6: $25000-$29999, 7: $30000-$34999, 8: $35000-$49999, 9: $50000-$74999, 10: > $75000.

### Statistical analysis

Risk aversion was estimated using a well-established approach commonly used in behavioral economics studies in which the index of risk aversion is derived using participants' responses on all 10 risk aversion questions [1,7,13]. Note that each question involves a binary choice between a gamble and a safe payoff. If subject *i *has a constant coefficient of relative risk aversion *γ_i_*, then the expected utility of the gamble for the *i *th participant at the *j*th question,$U_{ij}^{G}$, is defined by the following function,

$$U_{ij}^{G}=\frac{Los{s_{j}}^{1-\gamma_{i}}}{2\left(1-\gamma_{i}\right)}+\frac{Gai{n_{j}}^{1-\gamma_{i}}}{2\left(1-\gamma_{i}\right)}$$

where *Loss_j _*is the suboptimal outcome and *Gain_j _*is the winning outcome in the *j*th gamble. Given that the loss and gain outcomes are equally likely, each is weighted by 0.5. However, since the questions did not involve actual losses, the previous formula was further simplified as

$$U_{ij}^{G}=0.5\times \frac{Gai{n_{j}}^{1-\gamma_{i}}}{1-\gamma_{i}}$$

Similarly, the safe option payoff for *i *th participant at *j*th question has the expected utility

$$U_{ij}^{S}=\frac{Saf{e_{j}}^{1-\gamma_{i}}}{1-\gamma_{i}}$$

where *Safe_j _*is the safe gain for the *j*th question.

Let observed outcomes in the trials be *Y*, and the decision of choosing the gamble by *Y *= 1; we hypothesized that the probability *P*(*Y *= 1) depends on the difference between expected utility of the gamble and safe option $U_{ij}^{G}-U_{ij}^{S}$. The odds of choosing the gamble over safe option therefore was formulated as

$$\frac{P\left(Y=1\right)}{1-P\left(Y=1\right)}=e^{U_{ij}^{G}-U_{ij}^{S}}$$

If $U_{ij}^{G}-U_{ij}^{S}=0$, then a participant was indifferent between the gamble and safe options (i.e., odds of taking the gamble = 1 as in $\frac{P\left(Y=1\right)}{1-P\left(Y=1\right)}=1$), while a positive $U_{ij}^{G}-U_{ij}^{S}$ suggests that a participant favored the gamble (i.e., odds greater than 1), and vice versa. The risk aversion *γ_i _*was estimated using the above formula.

After computing risk aversion, we used a nonlinear mixed model to investigate whether variation in risk aversion *γ_i _*was associated with the covariates of interest [14-17]. The model was specified as follows,

$$\begin{array}{c} \text{logit}\left(P\left(Y_{ij}=1|X_{i},\gamma_{i}\right)\right)\\ =\frac{0.5\times \text{Gai}{\text{n}}^{1-f^{-1}\left(X_{i}\beta +\mu_{i}\right)}-\text{Saf}{\text{e}}^{1-f^{-1}\left(X_{i}\beta +\mu_{i}\right)}}{1-f^{-1}\left(X_{i}\beta +\mu_{i}\right)} \end{array}$$

Here **X**_*i *_was the covariate vector associated with unknown parameter vector **β**; *μ_i _*was the random effect associated with *i *th participant and was assumed to have a normal distribution with mean 0 and variance *σ*^2^; and the link function *f *= logit was introduced to ensure that risk aversion *γ_i _*estimates fall between 0 and 1.

The maximum likelihood estimates were derived to test the association between risk aversion and cognitive function, controlling for age, sex, education, and income. The risk aversion estimate for *i *th participant, ${\hat{\gamma }}_{i}$, was predicted using the parameter estimates $\hat{\beta }$ and empirical Bayes estimates of ${\hat{\mu }}_{i}$. All analyses were implemented in SAS version 9.2 using NLMIXED procedure [18].

## Results

### Descriptive properties of risk aversion

For descriptive purposes, Table 2 shows the distribution of participants who took the gamble for each risk aversion question. In the overall sample, about 50% of participants took the gamble when the potential gamble gain amount was double the safe payment amount. The percentage of participants who took the gamble remained at about 50% when the potential gain was quadruple the safe payment, and 79% of participants took the gamble when the potential gain amount was 10 times the safe payment.

**Table 2:** Percent of participants who took the gamble for each risk aversion question

Potential Gamble Gain Amount if Flip Heads	Whole sample Took gamble n, %	High cognition (top quartile) Took gamble n, %	Low cognition (bottom quartile) Took gamble n, %
$21.79	94, 26%	13, 15%	32, 34%
$22.64	83, 23%	14, 15%	26, 28%
$23.74	104, 28%	18, 19%	33, 35%
$25.19	123, 33%	26, 28%	34, 37%
$27.20	131, 36%	27, 29%	36, 39%
$30.18	194, 53%	53, 57%	44, 47%
$34.98	183, 50%	45, 48%	41, 44%
$43.88	184, 50%	52, 56%	35, 38%
$64.96	197, 53%	60, 65%	38, 41%
$151.19	290, 79%	78, 84%	65, 70%

Table 2 also shows the likelihood of taking the gamble among those in the upper versus lower quartiles of global cognitive function. Crude examination of these data suggests that persons with lower cognitive function were more likely to take the gamble compared to those with higher cognitive function when the potential gain was less than double the safe payment amount (less than $30). By contrast, those with lower cognitive function were less likely to take the gamble when the potential gamble gain doubled or more than doubled the safe payment amount. This suggests that those with lower cognitive function were less likely to make the optimal choice given the potential payments associated with the gambles. Equivalently, subjects with lower cognitive function were relatively less responsive to the changing incentives that they faced across the 10 risk aversion questions.

### Bivariate associations of risk aversion with demographic and contextual variables and global cognition

The mean estimate of risk aversion derived from participants' responses to all 10 questions was 0.394 (standard deviation (SD) = 0.314; range: 0.026 to 0.864), with higher values indicating greater risk aversion. Because little is known about the correlates of risk aversion in advanced age and because the correlates identified in studies of younger persons are inter-related, we first conducted correlation analyses to examine the bivariate associations between the demographic and contextual factors and cognition with risk aversion. In these analyses, we found that age was positively but not significantly associated with risk aversion (r = 0.09, p = 0.094). By contrast, education was negatively associated with risk aversion (r = -0.13, p = 0.01), and a simple t-test suggested that, on average, women were more risk averse than men (means for men and women were 0.28 and 0.43, respectively; *t *= 4.27, df = 367, *p*-value < .001). Income was negatively associated with risk aversion (r = -0.20, p < 0.001), such that persons with lower incomes tended to be more risk averse. Finally, global cognitive function was negatively associated with risk aversion (r = -0.17, p < 0.001), such that a lower level of cognitive function was associated with greater risk aversion.

### Multivariate associations and the link between global cognition and risk aversion

**Figure 1:**
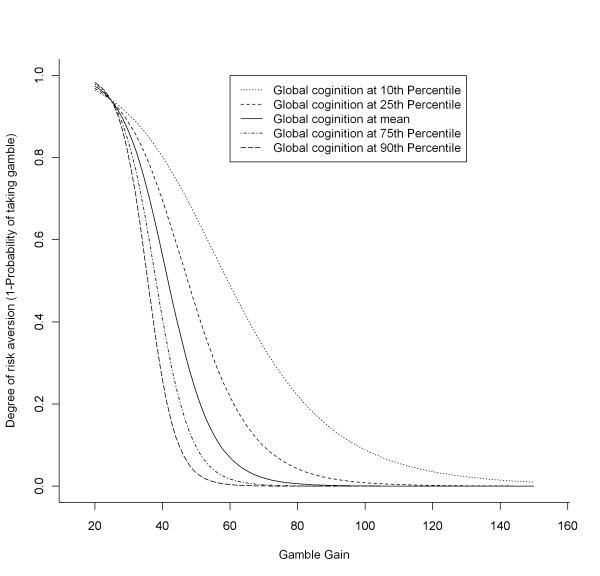
**Association of cognition with risk aversion as derived from a mixed effects model**. Results are illustrative of a typical participant (i.e., female with the median age, education, and income).

Next, we conducted a mixed effect model to test the hypothesis that cognitive function is associated with risk aversion; this model adjusted for age, sex, education, and income, and included a term for global cognition. In this core model, global cognitive function was related to risk aversion, such that a lower level of global cognitive function was associated with greater risk aversion (estimate = -1.05, SE = 0.34, p = 0.003; Table 3). Notably, however, sex was the only other covariate significantly associated with risk aversion (estimate = -1.49, SE = 0.39, p < 0.001), although income had a marginally significant inverse relationship with risk aversion (p = 0.052). Figure 1 shows the estimated probabilities of risk aversion as a function of the potential gamble gains stratified by different levels of global cognition; that is, from the top to bottom, the five curves project the likelihood of taking the safe payment (*i.e*., $15 for sure) by a typical female participant with the median income, 16 years of education, and 84 years of age, but at different percentiles of global cognition (10^th^, 25^th^, 50^th^, 75^th^, 90^th^, respectively). The figure presents a clear pattern whereby participants with lower global cognition were more risk averse (or less likely to take the gamble) compared to participants with higher global cognition, particularly after the potential gamble gain amount exceeded $30.

**Table 3:** Association of global cognitive function with risk aversion

Covariate	Estimate	SE	P-value
Age	0.021	0.024	0.392
Sex	-1.494	0.385	< 0.001
Education	-0.008	0.056	0.881
Income	-0.132	0.068	0.052
Global cognition	-1.048	0.344	0.003

*Estimated from a mixed effects model; note that females are coded as 0 and males as 1.

### Sensitivity analyses to examine the robustness of the association of global cognition with risk aversion

Importantly, although we excluded persons with dementia from these analyses, it is now widely recognized that dementia has a long preclinical phase during which many older persons exhibit cognitive decline but of insufficient severity to warrant a diagnosis of dementia. This stage is commonly referred to as mild cognitive impairment (MCI), and 79 (21%) persons included in the analyses above were classified as having MCI. Because it is possible that the association of cognition with risk aversion was influenced by their inclusion or differs among persons without any cognitive impairment compared to those with MCI, we repeated the core model described above with additional terms for MCI and the interaction of MCI with cognition. No interactions were found (data not shown), suggesting that the association of cognition with risk aversion did not differ along diagnostic lines.

Finally, to ensure that the finding was not driven by the influence of persons with MCI, we repeated the analysis described above again after excluding persons with MCI. The association between risk aversion and cognition persisted even among persons without cognitive impairment (data not shown).

### Domain specific analyses

In exploratory analyses aimed to determine whether the relation of cognition with risk aversion was of a general nature or reflected domain specific associations, we examined the association of five specific cognitive abilities with risk aversion via separate mixed effects models; these models adjusted for age, sex, education, and income and included a term for the relevant cognitive domain. In these analyses, semantic memory (estimate = -0.84, SE = 0.31, p = 0.007), episodic memory (estimate = -0.53, SE = 0.24, p = 0.032), working memory (estimate = -0.58, SE = 0.22, p = 0.010), and perceptual speed (estimate = -0.42, SE = 0.21, p = 0.05) were negatively associated with risk aversion; visuospatial abilities were not.

## Discussion

In a cohort of 369 community-based older persons free of dementia, we examined the correlates of risk aversion and tested the hypothesis that cognitive ability is related to risk aversion. Correlation analyses revealed links between sex, education, income, and cognition with risk aversion; however, the association between age and risk aversion was not significant. Further, the results of the mixed model suggested that, when considered together, only global cognitive function and sex were associated with risk aversion. Importantly, the association of cognitive ability with risk aversion was robust in that it persisted even when we considered the potential influence of persons exhibiting the earliest signs of cognitive impairment, a stage commonly referred to as MCI, and after excluding those with MCI. These findings are the first that we are aware of addressing the relation of cognitive ability with risk aversion among older persons with a wide spectrum of cognitive abilities and provide new data regarding the relative influence of cognitive ability versus related demographic and contextual factors on risk preferences. The finding that cognitive ability is related to risk aversion in advanced age may have important implications for improving financial and health decision-making, health behaviors, and ultimately well being among older persons.

In recent years, risk preferences have emerged as a topic of great interest in economics, behavioral economics, and neuroeconomics studies in part because of their strong association with real world economic and health outcomes [3,5,19,20]. The literature on risk aversion in aging is limited, however, consisting mostly of studies aimed to determine whether older persons are more or less risk averse than younger persons [21]. We are not aware of any study that has examined the relation of cognition with risk aversion among older persons or that simultaneously considered the potential influence of age, education, sex and income, despite their well known associations with both risk aversion and cognition. The lack of focus on risk aversion reflects an important gap in the aging literature, particularly given that older adults have to make several complex decisions just at a time when, for many, cognitive abilities are beginning to deteriorate. In fact, emerging data suggest that older persons often make suboptimal financial and other decisions as compared to younger persons [21,22]. The reasons why remain unknown, and risk preferences may represent an understudied yet potentially important factor. The present study identified cognitive ability and female sex as correlates of risk aversion in a diverse cohort of community based older persons and showed that the association of cognitive ability with risk aversion was robust among persons with a wide spectrum of cognitive function. If risk aversion is associated with real world decision making in aging as it is at younger ages, then these findings may suggest that older persons, particularly those with lower cognitive abilities, could benefit from assistance in understanding risk/benefit ratios and considering all possible options (not just the safe choice) when making consequential decisions.

Notably, in addition to education, sex, and income, some prior studies have reported associations between age and risk aversion, and the prevailing view seems to be that older age is associated with greater risk aversion [1,20-22]. In correlation analyses, education, sex and income were associated with risk aversion in directions consistent with findings in younger persons; however, age was not significant, possibly due to the restricted age range of our sample. Nevertheless, as we noted earlier, it has been difficult to disentangle the effect of cognition from the effects of other demographic and contextual (*e.g*., income) variables, which are related to both risk aversion and cognitive ability. The results of a mixed effect model suggest that, when the relevant correlates are considered simultaneously, only cognitive ability and sex are associated with risk aversion among older persons, with a trend for income. Thus, cognitive ability appears to be a robust determinant of risk aversion even in advanced age and among community based persons with a wide spectrum of cognitive function. Future research is needed to understand the trajectory of risk aversion in advanced age and to determine whether risk aversion changes over time with advancing age.

Although the association of cognitive ability with risk aversion has been examined in studies of younger persons, it remains unclear whether this is a general or domain-specific association either in younger or older persons [23]. In this study, performances on semantic memory, episodic memory, working memory, and perceptual speed were related to risk aversion; visuospatial abilities were not. These findings suggest that the association of cognition with risk aversion is of a fairly general nature. Further, it is noteworthy that the strongest association was observed for semantic memory, the domain of cognition associated with ideas and concepts and that represents acquired knowledge about the world. In fact, semantic memory is considered by some to serve as an indicator of crystallized cognitive ability; thus, the strong association between semantic memory and risk aversion is consistent with the results from at least one prior study that examined "intellectual ability" using two subscales from an intelligence test and reported a general association between "intelligence" and risk aversion among a large sample of German adults [5].

This study has a number of strengths, including the detailed assessment of risk aversion and cognition using standardized questions in a fairly large cohort of diverse community-dwelling older adults free of dementia. Additional strengths include the ability to examine the role of other important correlates and to conduct a number of sensitivity analyses to ensure that the findings were not due to the inclusion of persons with preclinical cognitive impairment. A limitation of the study is the selected nature of this volunteer cohort, which may have restricted our range of risk aversion and may limit the generalizability of findings. Another limitation was the assessment of risk aversion at a single point in time (rather than measuring change over time). Future studies are needed to investigate potential age-related changes in risk aversion and to examine the predictive association of risk aversion with financial and health decisions and outcomes in advanced age so that we may better understand how risk preferences impact real world outcomes across the lifespan.

## Conclusions

In a cohort of 369 community-based older persons free of dementia, we found that cognitive function was associated with risk aversion, a construct that has received little focus in studies of aging yet has been shown to be predictive of several important financial and health-related outcomes. Moreover, the association of cognition with risk aversion was robust in that it persisted even when we excluded persons exhibiting the very earliest manifestation of cognitive decline. These findings are the first that we are aware of addressing the relation of cognitive ability with risk aversion among older persons with a wide spectrum of cognitive abilities. Given that older persons are confronted with a host of complex, often risky, and very influential decisions just at a time when many face the ravages of cognitive decline, the association of cognition with risk aversion may have important implications for improving financial and health decision-making, health behaviors, and ultimately well being among older persons.

## Competing interests

The authors declare that they have no competing interests.

## Authors' contributions

PB had full access to all of the data and takes responsibility for the integrity of the data and the accuracy of the data analysis and affirms that everyone who contributed significantly to the work has been listed. She and DAB were involved with study concept and design, analysis and interpretation of data, and preparation of the manuscript. ASB, LY, DIL, and DAB critically reviewed the manuscript for important intellectual content, and LY performed the data analysis and assisted with interpretation of the data. All authors have seen and approved the final version.

## Pre-publication history

The pre-publication history for this paper can be accessed here:

http://www.biomedcentral.com/1471-2318/11/53/prepub
